# Comparative phylogeography of five widespread tree species: Insights into the history of western Amazonia

**DOI:** 10.1002/ece3.5306

**Published:** 2019-06-11

**Authors:** Eurídice N. Honorio Coronado, Kyle G. Dexter, Michelle L. Hart, Oliver L. Phillips, R. Toby Pennington

**Affiliations:** ^1^ Instituto de Investigaciones de la Amazonia Peruana Iquitos Peru; ^2^ School of GeoSciences University of Edinburgh Edinburgh UK; ^3^ Royal Botanic Garden Edinburgh Edinburgh UK; ^4^ School of Geography University of Leeds Leeds UK; ^5^ Department of Geography University of Exeter Exeter UK

**Keywords:** Amazon biogeography, genetic diversity, late successional species, phylogeography, pioneer species

## Abstract

Various historical processes have been put forth as drivers of patterns in the spatial distribution of Amazonian trees and their population genetic variation. We tested whether five widespread tree species show congruent phylogeographic breaks and similar patterns of demographic expansion, which could be related to proposed Pleistocene refugia or the presence of geological arches in western Amazonia. We sampled *Otoba parvifolia/glycycarpa* (Myristicaceae), *Clarisia biflora*, *Poulsenia armata*, *Ficus insipida* (all Moraceae), and *Jacaratia digitata* (Caricaceae) across the western Amazon Basin. Plastid DNA (*trn*H–*psb*A; 674 individuals from 34 populations) and nuclear ribosomal internal transcribed spacers (ITS; 214 individuals from 30 populations) were sequenced to assess genetic diversity, genetic differentiation, population genetic structure, and demographic patterns. Overall genetic diversity for both markers varied among species, with higher values in populations of shade‐tolerant species than in pioneer species. Spatial analysis of molecular variance (SAMOVA) identified three genetically differentiated groups for the plastid marker for each species, but the areas of genetic differentiation were not concordant among species. Fewer SAMOVA groups were found for ITS, with no detectable genetic differentiation among populations in pioneers. The lack of spatially congruent phylogeographic breaks across species suggests no common biogeographic history of these Amazonian tree species. The idiosyncratic phylogeographic patterns of species could be due instead to species‐specific responses to geological and climatic changes. Population genetic patterns were similar among species with similar biological features, indicating that the ecological characteristics of species impact large‐scale phylogeography.

## 1 INTRODUCTION

Comparative phylogeography has the potential to elucidate the demographic history of co‐occurring species at large spatial scales since different organisms are expected to accumulate similar geographic genetic signatures in response to common historical biogeographic drivers (Avise et al., 1987). For example, congruent geographic patterns of genetic diversity and divergence among populations in different tree species have been related to the occurrence of Pleistocene refugia and postglacial colonization events, in both temperate vegetation in the northern hemisphere (Petit et al., 2003) and tropical areas in Central Africa (Dauby et al., 2014). In the Neotropics, comparative phylogeographic studies of tree species have focused on relating common phylogeographic breaks to geological events, such as the rise of the Andes (Dick, Lewis, Maslin, & Bermingham, 2013; Jones, Cerón‐Souza, Hardesty, & Dick, 2013) or the boundary of Pliocene islands in Central America (Poelchau & Hamrick, 2013). In western Amazonia, common patterns of genetic differentiation among communities of *Inga* species (Fabaceae) were reported across a 250‐km transect, which were used to argue for a zone of secondary contact between two historically isolated communities that were fragmented by a common event (Dexter, Terborgh, & Cunningham, 2012).

Early phylogeographic studies of animals in the western Amazon basin showed that phylogeographic breaks coincided with geological paleoarches such as the Iquitos Arch in the Jurua River (Lougheed, Gascon, Jones, Bogart, & Boag, 1999; da Silva & Patton, 1998). In plants, paleoarches such as the Iquitos and Fitzcarrald arches also correlated well with some of the genetic groups detected in microsatellite markers for *Theobroma cacao* (Malvaceae), a widespread tree species in the Amazon (Motamayor et al., 2008), and the Fitzcarrald Arch coincided with the common phylogeographic break identified for a range of *Inga* tree species by Dexter et al. (2012). Paleoarches in western Amazonia, such as the Fitzcarrald Arch, formed because of uplift of the Andean Cordillera during the Late Miocene/Pliocene (Räsänen, Salo, Jungnert, & Pittman, 1990). These geological features may have not attained sufficient elevation to directly isolate populations of lowland species (de Fátima Rossetti, Mann de Toledo, & Góes, 2005). Rather, the uplift of these paleoarches caused significant changes in drainage systems creating new habitats and environmental conditions (Espurt et al., 2010; Higgins et al., 2011; Regard et al., 2009), which may have played a role in shifting the distribution of species and causing population isolation.

Pleistocene climatic oscillations and contraction of the Amazon rain forest into refuges is another process that could cause common phylogeographic patterns across rain forest tree species (Prance, 1982). Past fragmentation of the rain forest is expected to leave a congruent genetic signature across lowland rain forest species, with refugial areas maintaining old populations with high levels of intraspecific genetic diversity, while populations in recently colonized areas would have low levels of genetic diversity (Dauby et al., 2014; Petit et al., 2003). Although the existence of such refuges for lowland species in Amazonia has been suggested to be controversial (Colinvaux, De Oliveira, & Bush, 2000), more recent studies indicate considerable impacts of glacial climates on Amazonia (Anhuf et al., 2006; Arruda, Fernandes‐Filho, Solar, & Schaefer, 2017; Cheng et al., 2013), for example, suggesting expansion of drier formations in eastern Amazonia. These studies indicate greater stability of forest in northwestern Amazonia, differing from earlier work (e.g., Prance, 1982), which suggested multiple separate refugia in northwestern Amazonia.

If rain forest tree species experienced shifts in their distribution during Pleistocene climatic oscillations, then we may also expect them to show similar patterns of population expansion in the same areas. The reconstruction of tropical rain forest distribution during the Last Glacial Maximum (LGM) based on paleoenvironmental data suggests reduction and fragmentation of humid forest in 54% of the current area in Amazonia (Anhuf et al., 2006). This study showed differences of climatic conditions across western Amazonia during the LGM, with more stable climates in northwestern Amazonia (NWA), and the expansion of vegetation adapted to dry conditions (dry forest/savanna) in peripheral areas, including southwestern Amazonia (SWA). Palynological studies also suggest the recent expansion of the Amazon rain forest in SWA (Mayle, Burbridge, & Killeen, 2000), which would be consistent with Amazonian expansion during the current, wetter, interglacial.

The potential of paleoarches and Pleistocene refugia to drive common phylogeographic patterns across multiple tree species has not been tested at a large spatial scale across the western Amazon basin. Given the independent nature of the sampling schemes and different spatial scales of previous studies (Dexter et al., 2012; Dick et al., 2013; Jones et al., 2013; Motamayor et al., 2008), they are hard to compare. Here, we sought to apply a standardized approach for multiple Amazon tree species to facilitate more robust comparisons among different species and populations to explore the importance of historical events in shaping common phylogeographic patterns. By employing uniform and thorough geographic sampling of populations across widespread western Amazonian tree species, including three late successional species and two pioneers, we make a comparative study of their genetic diversity and population genetic structure. In particular, using plastid and nuclear markers sampled in 34 locations across 2,500 km of geographic distance, we test:

1. If there are concordant areas of genetic differentiation across populations of the five tree species, which would suggest common underlying reasons for phylogeographic congruence, due either to a) geological paleoarches such as the Fitzcarrald Arch or b) the contraction of populations into refugia during Pleistocene climate cycles.
2. If species show a genetic signature of population expansion in the same geographic areas, and particularly in southwestern Amazonia.

## 2 MATERIALS AND METHODS

### 2.1 The study species

We studied five widespread tree species that have overlapping ranges in western Amazonia. *Otoba parvifolia/glycycarpa* (Myristicaceae), *Clarisia biflora* Ruiz & Pav., and *Poulsenia armata* (Miq.) Standl. (both Moraceae) are shade‐tolerant species characteristic of primary forest, while *Ficus insipida* Willd. (Moraceae) and *Jacaratia digitata* (Poepp. & Endl.) Solms (Caricaceae) are light‐demanding pioneer species that tend to grow in secondary forest. Pollen of these species is dispersed by different insects, and seeds are dispersed by animals (Table 1). These species were chosen because they are abundant in western Amazonia and are readily distinguished from congeneric species, thereby facilitating collection and correct identification of samples in the field. One taxonomically difficult exception was *O. parvifolia/glycycarpa* (Markgr.) A.H. Gentry that was not consistently distinguished morphologically from *O. glycycarpa* (Ducke) W. Rodrigues & T.S. Jaramillo. These two species were also not distinguished genetically using *trn*H‐*psb*A sequences (E.N. Honorio, unpublished data). Here, they are treated as one group of samples (*O. parvifolia/glycycarpa*) because both genetic evidence and morphological evidence suggest that they are the same species. The phylogeographies of four species are characterized in this study for the first time, while the phylogeographic patterns of *F. insipida* were previously published by Honorio Coronado et al. (2014) and da Costa et al. (2017).

**Table 1:** Ecological characteristics of the five studied western Amazonia tree species

Species (Family)	Floral sexuality^a^	Pollen dispersa1^a^	Seed dispersal	Geographic distribution
*Otoba parvifolia/glycycarpa*(MYRISTICACEAE)	Dioecious	Insects (beetles?)	Vertebrates (bats)	Central America, northern Andes and western Amazonia
*Clarisia biflora* (MORACEAE)	Dioecious	?	Vertebrates?	Mesoamerica, northern Andes and western Amazonia
*Poulsenia armata* (MORACEAE)	Monoecious^b^	Insects (thrips)	Vertebrates (bats)	Mesoamerica, northern Andes and western Amazonia
*Ficus insipida* (MORACEAE)	Monoecious^c^	Insects (wasps)	Vertebrates (fish, bats)	Mesoamerica, northern Andes and western Amazonia
*Jacaratia digitate* (CARICACEAE)	Dioecious	Insects (moths)	Vertebrates (monkeys, large birds)	Western Amazonia

a Bawa, Bullock, et al. (1985); Bawa, Perry, et al. (1985).

b Separate male and female inflorescences.

c Male and female flowers are inside enclosed receptacles called syconia.

### 2.2 Sampling strategy

**Figure 1:**
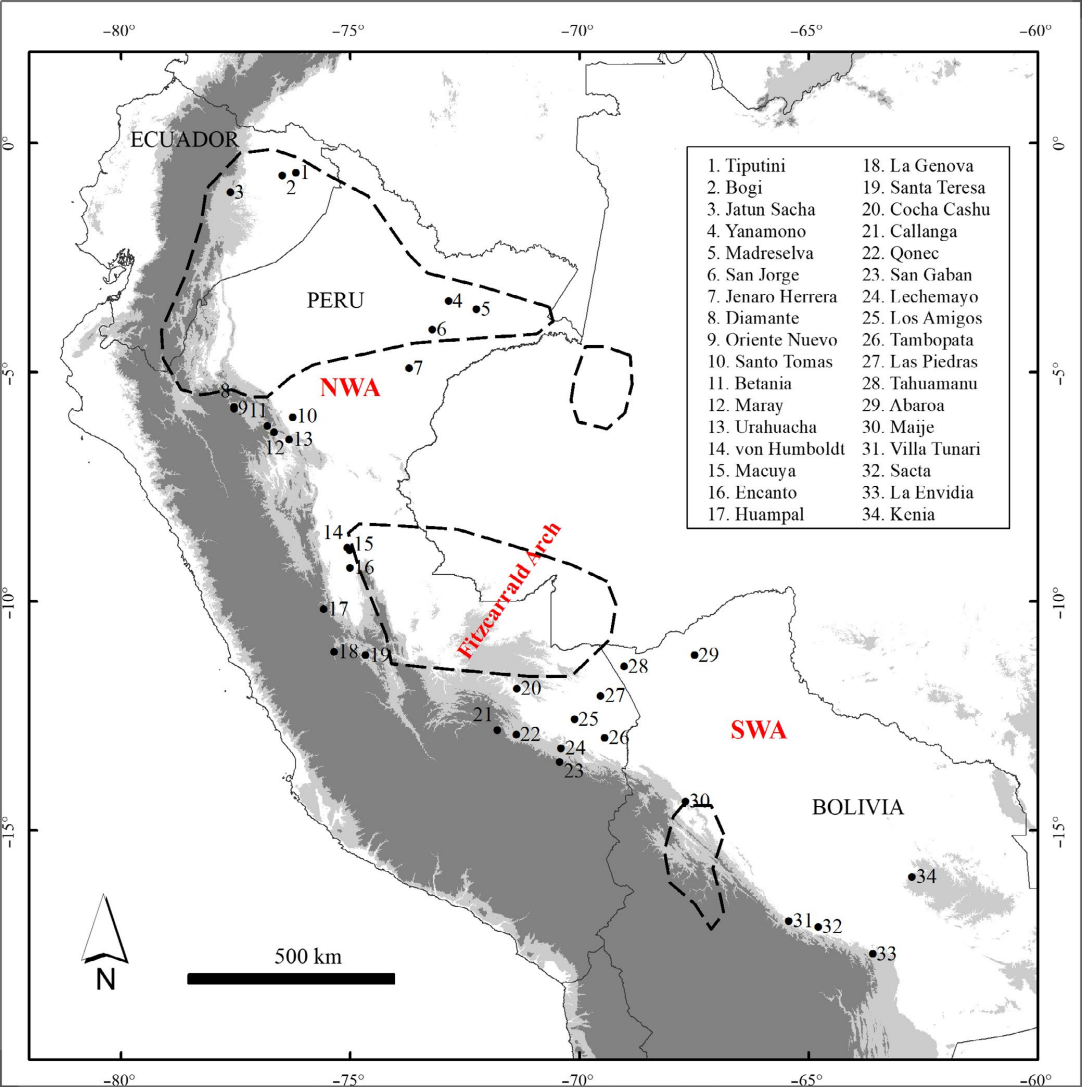
Map with the location of sampled localities, the Fitzcarrald Arch and the two regional groups (NWA, northwest Amazon and SWA, southwest Amazon). Areas proposed as refugia for plants by Prance (1982) are indicated in dotted lines. The Andean Cordillera is indicated by low altitudes in light gray (400–1200 m.a.s.l.) and higher altitudes in dark gray

Samples were collected in regions where these species occur in western Amazonia, giving priority to sites where at least three were previously reported. We sampled 34 different sites in Ecuador, Peru, and Bolivia (Figure 1), with 22 sites sampled for *O. parvifolia/glycycarpa*, 19 for *C. biflora*, 12 for *P. armata*, 23 for *F. insipida,* and 17 for *J. digitata*. Sample sizes per population ranged from 1 to 19 (mean = 7 individuals). For each individual collected, leaf samples were dried and stored in silica gel. All individuals were photographed in the field, and at least one herbarium voucher was collected from each population to allow subsequent verification of identification. These vouchers were collected by E. Honorio, K. Dexter, and A. Monteagudo and deposited at LOJA in Ecuador, HOXA and MOL in Peru, and UAP and USZ in Bolivia.

Our sampling spans the Fitzcarrald Arch (FA), a geological feature that divides the Amazonia into a northern and southern foreland basin (Espurt et al., 2010). Sampling on either side of the FA was equivalent in terms of number of populations sampled, and their geographic extent (107,233 km^2^ north of FA vs. 130,554 km^2^ south of FA), and the mean geographic distance between sampled populations did not differ significantly between the two regions (*t* test: *t* = 1.51, *df *= 443.4, *p* = 0.13). We therefore chose the FA to divide the sampled populations into two regional groups for some of the downstream analyses. The area north of the FA is generally termed the northwest Amazon (NWA) and is thought to have experienced a stable climate through Pleistocene climatic oscillations (Anhuf et al., 2006), while the area south of the FA is generally termed the southwest Amazon (SWA) and is thought to have experienced recent rain forest expansion (Mayle et al., 2000).

### 2.3 DNA extraction and sequencing

Total genomic DNA was extracted using the CTAB method (Doyle & Doyle, 1987) for most species, while the DNeasy® Plant Mini Kit (Qiagen) was used for *O. parvifolia/glycycarpa*. Seven plastid markers were tested for amplification and sequencing: *rpl*32*‐trn*L*, trn*Q‐5'‐*rps*16, 3'*trn*V‐*nd*HC, *atp*I‐*atp*H, *trn*D‐*trn*T, *trn*H‐*psb*A, and *trn*L‐*trn*F (Shaw, Lickey, Schilling, & Small, 2007). The noncoding marker *trn*H*‐psb*A was chosen because of its high amplification success and variability among populations for all species. In addition, the internal transcribed spacer (ITS) of the nuclear ribosomal DNA was amplified and sequenced using the ITS1 and ITS4 primers (White, Bruns, Lee, & Taylor, 1990). PCRs followed the conditions described for *F. insipida* in Honorio Coronado et al. (2014). PCR products were visualized via 1% agarose gel electrophoresis, and products were purified using ExoSAP‐IT (USB Corporation). Cycle sequencing was conducted in 10 μl solutions containing 3 μl PCR product, 0.5 μl of BigDye (Applied Biosystems), 2 μl sequencing reaction buffer 5×, 0.32 μl of primer, and 4.18 μl of distilled H_2_O. All forward and reverse strands were edited in Sequencher v. 5.0 (Gene Codes Corporation) and nucleotide substitutions, indels (i.e., insertions or deletions), and inversions were visually checked against the original electropherograms. The alignment was created manually in Mesquite v. 2.74 (Maddison & Maddison, 2001). Mononucleotide repeat polymorphisms were excluded from all subsequent analyses.

### 2.4 Genetic diversity and haplotype definition

Genetic variation in each species was recorded as the number of nucleotide substitutions, indels, and inversions, the number of* trn*H*‐psb*A haplotypes and ITS ribotypes, and the number of private haplotypes and ribotypes (i.e., the number of haplotypes or ribotypes unique to each population). Genetic diversity was computed independently for each species using the *trn*H*‐psb*A and ITS sequences by estimating the probability that two individuals carry distinct haplotypes or ribotypes (h) and the mean genetic distance between haplotypes or ribotypes of two distinct individuals (v) averaged over populations (*h*_S_ and *v*_S_) and over all population pairs (*h*_T_ and *v*_T_) in Permut CpSSR 2.0 (Pons & Petit, 1996). Measures were obtained by a permutation procedure set to 10,000 replicates.

For each species, haplotypes and ribotypes and their genealogical relationships were estimated independently for *trn*H*‐psb*A and ITS sequences using statistical parsimony in the package “pegas” (Paradis, 2010) in the R Statistical Software v. 2.15.2. Individual indels and inversions were treated as single mutation events. We mapped the distribution of haplotypes and ribotypes using ArcMap v. 10.3.

### 2.5 Differentiation and population genetic structure

A pattern of isolation by distance was assessed independently for *trn*H*‐psb*A and ITS sequences by Mantel test using the package “adegenet” in the R Statistical Software. Specifically, we compared overall Nei's pairwise genetic distance among populations and the logarithm of the Euclidean geographic distances. The significance of the relationship was tested with 10,000 permutations. Global genetic differentiation between populations was assessed by computing *G*_ST_ (= 1−*h*_S_/*h*_T_) that makes use only of haplotype or ribotype frequency and *N*_ST_ (=1−*v*_S_/*v*_T_), for which sequence similarities between the haplotypes or ribotypes are taken into account. The presence of phylogeographic signal was assessed by testing whether *N*_ST_ was significantly higher than *G*_ST_ based on 10,000 permutations in Permut CpSSR 2.0 (Pons & Petit, 1996).

A spatial analysis of molecular variance (SAMOVA) was performed for each species for *trn*H*‐psb*A and ITS sequences using Samova v. 1.0 (Dupanloup, Schneider, & Excoffier, 2002) to determine the position of genetic breaks among populations. Several runs were performed using increasing numbers of groups (k = 1−20) and 100 annealing simulations for each k. In each, populations were clustered into genetically and geographically homogenous groups (Dupanloup et al., 2002). The number of groups was chosen so as to maximize genetic differentiation among them (Φ_CT_). Genetic structure using pairwise nucleotide differences was further examined by analysis of molecular variance in Arlequin v. 3.5 (Excoffier & Lischer, 2010) for all populations and for groups of populations defined by SAMOVA. Significance of genetic structure indices was tested using a nonparametric randomization procedure.

### 2.6 Historical demographic analysis

We tested for patterns of population expansion independently for each species using *trn*H*‐psb*A and ITS sequences for all populations. We used the neutrality test, Fu's Fs (Fu, 1997), which is based on the number of pairwise differences among haplotypes or ribotypes. Significant positive values indicate population contraction and balancing selection that leads to an excess of intermediate frequency types, while negative values indicate population expansion, directional selection, or presence of weakly deleterious mutations, all of which lead to an excess of rare variants. In addition, we simulated the distribution of the pairwise differences between haplotypes under a sudden demographic expansion model (Schneider & Excoffier, 1999). Multimodal distributions generally indicate that populations are at demographic equilibrium and unimodal ones suggest a recent demographic expansion (Rogers & Harpending, 1992). Statistics to compare the observed and simulated distributions were based on Harpending's raggedness index (HRI) using 1,000 parametric bootstrap replicates (Harpending, 1994). All tests were performed in Arlequin v. 3.5 (Excoffier & Lischer, 2010).

### 2.7 Regional patterns

The number of haplotypes/ribotypes and private haplotypes/ribotypes, overall genetic diversity (*v*_T_), and demographic analyses were estimated for each geographic region by independently analyzing populations located in the NWA and SWA regions. The regional analyses were only performed for *trn*H*‐psb*A sequences, because of data availability.

## 3 RESULTS

### 3.1 Genetic diversity and haplotype definition

A total of 674 individuals were sequenced for the *trn*H‐*psb*A marker and 214 individuals for ITS. Total length of the regions varies among species from 333 to 527 base pairs for *trn*H‐*psb*A and from 607 to 652 bp for ITS (Table 2). We failed to obtain ITS sequences for *O. parvifolia/glycycarpa*.

**Table 2:** Number of samples, number of haplotypes or ribotypes, genetic diversity (*h*_T_ and *v*_T_), mantel test, genetic differentiation (*G*_ST_ and *N*_ST_), and demographic analyses (Fu's Fs and HRI) for the *trnH‐psbA* and ITS sequences in five western Amazonia tree species. Note that *p*‐values for Fu's Fs are only considered significant at the 95% level if the *p*‐value is lower than 0.02 (Excoffier & Lischer, 2010)

Taxon	*N*	Pops	Ltot	L	NS	IV	ID	Hap/Rib	Pr	*h*_S _(SE)	*v*_S _(SE)	*h*_T _(SE)	*v*_T _(SE)	Mantel r (*p*‐value)	*G*_ST _(SE)	*N*_ST _(SE)	*p*‐value	Fu's Fs (*p*‐value)	HRI (*p*‐value)
*trn*H‐*psb*A																			
*O. parvifolia/glycycarpa*	186	22	333	316	19	1	0	16	8	0.10 (0.04)	0.05 (0.02)	0.94 (0.03)	0.94 (0.13)	0.11 (N.S.)	0.89 (0.04)	0.95 (0.02)	<0.01	0.01 (0.55)	0.09 (0.00)
*C. biflora*	124	19	416	384	13	1	1	16	11	0.13 (0.05)	0.10 (0.05)	0.98 (0.02)	0.98 (0.08)	0.25 (<0.05)	0.87 (0.05)	0.90 (0.05)	N.S.	−2.62 (0.20)	0.04 (0.14)
*P. armata*	61	12	381	363	10	1	0	7	2	0.16 (0.08)	0.03 (0.02)	0.87 (0.05)	0.89 (0.04)	0.05 (N.S.)	0.82 (0.09)	0.97 (0.02)	<0.05	4.45 (0.93)	0.07 (0.19)
*F. insipida*	182	23	375	340	8	1	3	12	7	0.34 (0.06)	0.27 (0.07)	0.72 (0.06)	0.72 (0.15)	0.18 (N.S.)	0.53 (0.09)	0.62 (0.09)	<0.05	−1.45 (0.34)	0.05 (1.00)
*J. digitata*	121	17	527	374	21	4	7	6	3	0.02 (0.02)	0.00 (0.00)	0.58 (0.14)	0.58 (0.23)	0.44 (<0.01)	0.96 (0.04)	1.00 (0.00)	N.S.	14.32 (1.00)	0.14 (1.00)
ITS																			
*C. biflora*	76	19	652	648	19	0	1	27	17	0.72 (0.07)	0.45 (0.06)	0.94 (0.03)	0.95 (0.06)	0.09 (N.S.)	0.23 (0.06)	0.53 (0.08)	<0.01	−12.79 (0.00)	0.01 (0.94)
*P. armata*	49	12	631	625	13	1	2	7	4	0.15 (0.08)	0.15 (0.13)	0.83 (0.08)	0.83 (0.30)	−0.03 (N.S.)	0.82 (0.10)	0.82 (0.13)	N.S.	3.66 (0.93)	0.06 (0.45)
*F. insipida*	77	23	635	635	1	0	2	4	2	0.15 (0.06)	0.15 (0.06)	0.14 (0.05)	0.14 (0.06)	−0.04 (*N.S*.)	N.A.	N.A.	N.A.	−2.19 (0.04)	0.72 (0.75)
*J. digitata*	12	11	607	607	3	0	0	3	1	N.A.	N.A.	N.A.	N.A.	N.A.	N.A.	N.A.	N.A.	0.65 (0.62)	0.28 (0.22)

Abbreviations: Hap/Rib, haplotypes or ribotypes; ID, insertions/deletions; IV, inversions; L, length of coded sequences; Ltot, total length of alignment; N, number of individuals; NS, nucleotide substitutions; Pops, number of populations; Pr, private haplotypes or ribotypes; SE, standard error.

**Figure 2:**
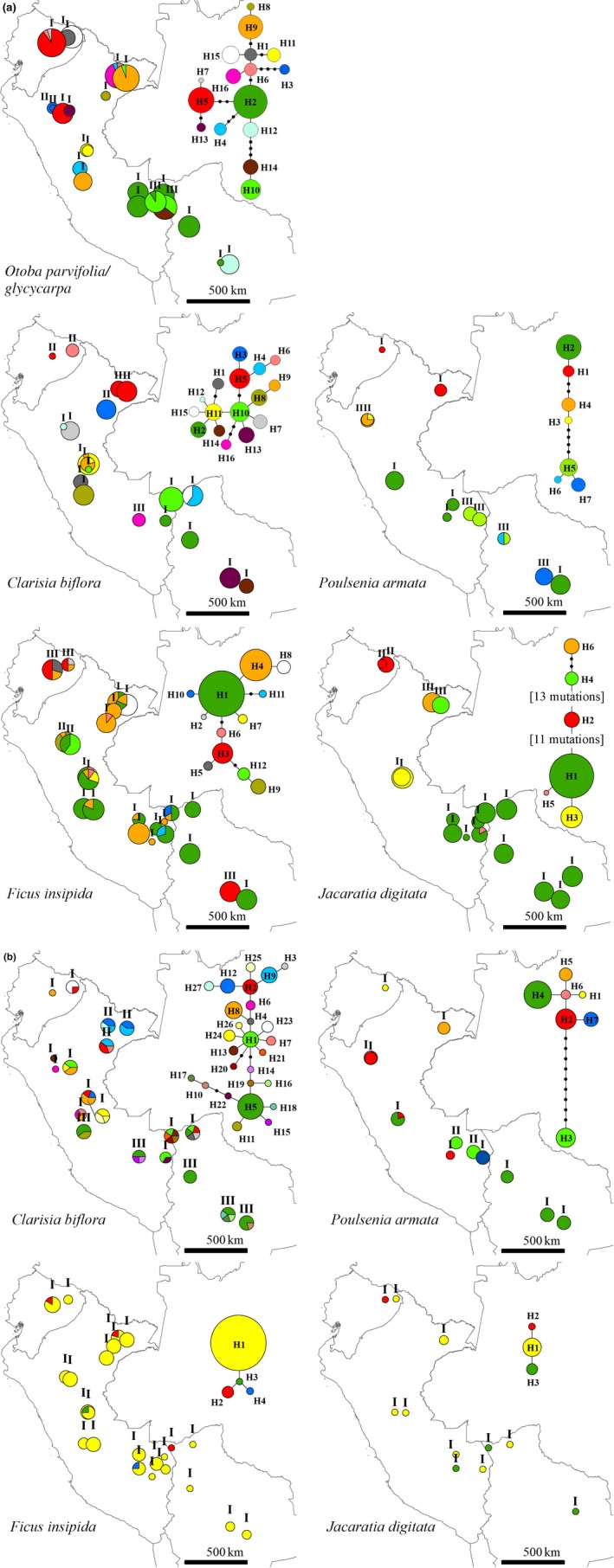
Haplotype and ribotype maps of five widespread species sampled in western Amazonia for (a) *trn*H*‐psb*A and (b) ITS sequences. Colors represent haplotypes or ribotypes, and these are not shared among species. The size of the circles is proportional to sample size for each population. Statistical parsimony networks of haplotypes or ribotypes are included for each taxon: Each link represents a single mutation, and black dots indicate missing haplotypes. Population labels indicate the SAMOVA group (I‐III)

For *trn*H‐*psb*A, 16 haplotypes were defined in *O. parvifolia/glycycarpa* and *C. biflora*, 12 in *F. insipida*, seven in *P. armata*, and six in *J. digitata* (Table 2; Figure 2a). Most species have at least 50% of haplotypes unique to a given population (private haplotypes), except for *P. armata* for which nearly all haplotypes occurred in two or more populations (Table 2). Overall diversity values for *trn*H‐*psb*A sequences, estimated by *h*_T_ and *v*_T_, were high in *O. parvifolia/glycycarpa*, *C. biflora,* and *P. armata* (over 0.87), slightly lower in *F. insipida* (0.71), and much lower in *J. digitata* (0.57) (Table 2).

For ITS, 27 ribotypes were defined in *C. biflora*, seven in *P. armata*, four in *F. insipida*, and three in *J. digitata* (Table 2; Figure 2b). 50% of ribotypes were private to a given population in *C. biflora* and *F. insipida* (Table 2). Overall diversity values for ITS sequences, estimated by *h*_T_ and *v*_T_, were high in *C. biflora* and *P. armata* (over 0.80), much lower in *F. insipida* (0.14), while measures were not available for *J. digitata* because it showed no intrapopulation genetic variation for ITS (Table 2).

### 3.2 Differentiation and population genetic structure

For *trn*H‐*psb*A, the Mantel test between genetic and geographic distances showed significant isolation by distance for only two species, *C. biflora* and *J. digitata* (Table 2). *Otoba parvifolia/glycycarpa* had highly divergent haplotypes and significant phylogeographic structure (Table 2). The most divergent populations were located in northern (haplotype 3) and southern Peru (haplotypes 10 and 14). *Clarisia biflora* had a group of closely related haplotypes, and most populations had single haplotypes. *Poulsenia armata* showed significant phylogeographic structure (Table 2) with the most divergent region occurring in southern Peru and Bolivia (H5, H6, and H7). *Ficus insipida* showed significant phylogeographic structure (Table 2) although haplotypes were not highly divergent. In this species, two haplotypes (H1 and H4) were widely distributed in most populations. *Jacaratia digitata* displayed highly divergent haplotypes among which one (H1) was widely distributed in all populations of southern Peru and Bolivia. In this species, populations in Ecuador and northern Peru hosted the most divergent haplotypes (Figure 2a).

For ITS, the Mantel test between genetic and geographic distances did not show significant isolation by distance for any species (Table 2). *Clarisia biflora* had significant phylogeographic structure (Table 2), and three or more ribotypes were present in most populations. The most divergent regions were located in northern Peru and close to the Andes from central Peru to Bolivia (Figure 2b). *Poulsenia armata* had only one highly divergent ribotype (H5) present in populations of southern Peru. *Jacaratia digitata* and *F. insipida* displayed closely related ribotypes, which were widely distributed in all populations (Figure 1b).

Genetic differentiation among populations for the *trn*H‐*psb*A sequences was generally above 91% in all species, except for *F. insipida* where only 67% of differentiation was explained by interpopulation nucleotide differences (Table 3). In the case of ITS, only *C.  biflora* (55%) and *P. armata* (96%) showed significant genetic differentiation among populations (Table 3). The SAMOVA defined three groups of populations for all species for the *trn*H‐*psb*A sequences (Figure 2a), with values of genetic differentiation (Φ_CT_) of 0.68 for *F. insipida*, 0.97 for *J. digitata*, 0.46 for *C. biflora*, 0.92 for *P. armata*, and 0.61 for *O. parvifolia/glycycarpa*. For ITS, *F. insipida* and *J. digitata* showed no geographic genetic structure (Figure 2b), while three groups were defined for *C. biflora* (Φ_CT_ = 0.61) and two groups for *P. armata* (Φ_CT_ = 0.92).

**Table 3:** Analyses of molecular variance based on pairwise nucleotide differences for the *trnH‐psbA* and ITS sequences in five western Amazonia tree species. The analysis to examine genetic structure was run using pairwise nucleotide differences and independently  for all populations and for populations grouped by SAMOVA groups

Taxa	All populations		SAMOVA groups			
	Among pops (%)	Within pops (%)	*N* groups	Among groups (%)	Among pops within groups (%)	Within pops (%)
*trn*H‐*psb*A						
*O. parvifolia/glycycarpa*	92.44**	7.56^N.S.^	3	60.71**	35.00**	4.29**
*C. biflora*	91.20**	8.80^N.S.^	3	46.15**	47.31**	6.54**
*P. armata*	97.85**	2.15^N.S.^	3	92.05**	6.44**	1.51**
*F. insipida*	66.94**	33.06^N.S.^	3	66.82**	13.01**	20.17**
*J. digitata*	99.79**	0.21^N.S.^	3	96.83**	3.08**	0.08**
ITS						
*C. biflora*	55.28**	44.72^N.S.^	3	60.76**	3.79**	35.45**
*P. armata*	96.28**	3.72^N.S.^	2	90.92**	7.63**	1.45**
*F. insipida*	6.99^N.S.^	93.01^N.S.^	1	N.A.	N.A.	N.A.
*J. digitata*	N.A.	N.A.	1	N.A.	N.A.	N.A.

Abbreviations: N.A., not applicable; N.S., not significant

* *p* < 0.05.

** *p* < 0.005.

### 3.3 Historical demographic analysis

Neutrality tests for the *trn*H‐*psb*A and ITS sequences were not significant in most species (*p* > 0.02; Table 2), except for *C. biflora* and marginally for *F. insipida*, both for ITS. A unimodal mismatch distribution was only observed in *C. biflora* for *trn*H‐*psb*A and ITS. However, based on the HRI statistics, the recent demographic expansion model was consistently accepted for all species and markers, except for *O. parvifolia/glycycarpa* (*p* > 0.05; Table 2).

### 3.4 Regional patterns

For *trn*H‐*psb*A, NWA and SWA regions had haplotypes private to each region in all species, but other haplotypes were present in both regions such as H10 in *O. parvifolia/glycycarpa*, H4 in *C. biflora*, and H1–H3 in *F. insipida* (Figure 2a). Overall genetic diversity was similar in both regions for *C. biflora* and *P. armata*, while diversity in NWA was much greater than in SWA in *J. digitata* and slightly greater in *O. parvifolia/glycycarpa* and *F. insipida* (Table 4). We had only marginally significant negative values of Fu's Fs for *J. digitata* in the SWA region. Within each regional group, recent expansion was consistently accepted for the SWA region for all species and for *C. biflora*, *P. armata,* and *F. insipida* in NWA (Table 4).

**Table 4:** Number of haplotypes and private haplotypes, genetic diversity, and demographic estimates for *trnH‐psbA* sequences in five western Amazonian tree species for each geographic region

Region	*O. parvifolia/glycycarpa*	*C. biflora*	*P. armata*	*F. insipida*	*J. digitata*
*N*° samples/*N*° populations					
NWA	108/14	71/12	23/5	112/12	42/6
SWA	78/8	53/7	38/7	70/11	79/11
*N*° haplotypes/*N*° private haplotypes					
NWA	13/12	10/9	4/3	10/7	4/4
SWA	4/3	7/6	4/3	5/2	2/2
Overall genetic diversity *v*_T ± _SE					
NWA	0.96 ± 0.13	0.96 ± 0.10	0.81 ± 0.18	0.81 ± 0.17	0.90 ± 0.05
SWA	0.67 ± 0.21	0.95 ± 0.19	0.93 ± 0.07	0.60 ± 0.25	0.03 ± 0.03
Neutrality test Fu's Fs (*p*‐value)					
NWA	−0.24 (0.52)	0.03 (0.59)	2.27 (0.88)	−0.16 (0.52)	21.51 (1.00)
SWA	5.37 (0.96)	0.94 (0.74)	7.32 (0.99)	0.87 (0.70)	−2.02 (0.03)
Mismatch distribution HRI (*p*‐value)					
NWA	0.14 (0.00)	0.06 (0.09)	0.15 (0.51)	0.04 (0.77)	0.25 (0.00)
SWA	0.15 (0.31)	0.06 (0.30)	0.17 (0.60)	0.11 (0.60)	0.90 (0.93)

## 4 DISCUSSION

### 4.1 No concordant areas of genetic differentiation

Across five Amazonian rain forest tree species, we did not find evidence for shared phylogeographic break that could have been congruent with either geological paleoarches or putative Pleistocene refugia. Of the various geological paleoarches in the Amazon (Motamayor et al., 2008), our sampling was best suited to testing the influence of the Fitzcarrald Arch on phylogeographic patterns, but we failed to find congruent genetic differentiation associated with this feature. This contrasts with the study of *Inga* species by Dexter et al. (2012), but it is important to note that only 10 of the 20 *Inga* species in Dexter et al. (2012) showed this genetic break. The Fitzcarrald Arch does not seem to represent a consistent historical or present‐day barrier to seed dispersal or gene flow for the Amazonian tree species in our study.

Recent work suggests stability of forest across northwestern Amazonia at the Last Glacial Maximum (Anhuf et al., 2006; Arruda et al., 2017; Cheng et al., 2013), which contrasts with earlier suggestions (see various chapters in Prance, 1982) of multiple refugia in this area. If there have been such multiple refugia, we would expect species to have congruent phylogeographic breaks in the areas between them. We found no such evidence for congruent phylogeographic breaks across the western Amazon basin. Our results contrast with those of a comparative phylogeographic study in tropical areas of Atlantic Central Africa that found congruent genetic patterns in five of eight tree study species supporting a scenario of past forest fragmentation caused by glacial climate change (Dauby et al., 2014). By using four densely sampled species, Heuertz, Duminil, Dauby, Savolainen, and Hardy (2014) also found a common genetic break with differentiation of northern and southern populations in the same region of Central Africa. Our contrasting results may indicate that there has been greater stability of Pleistocene climates in western Amazonia than in tropical Africa.

For each of the five tree species in our study, we found significant genetic differentiation among populations for the plastid marker, with clear geographic breaks between more genetically homogeneous sets of populations, but these breaks are not geographically coincident. Genetic breaks found in western Amazonia could represent areas of secondary contact of previously differentiated lineages put in contact by subsequent dispersal events. Moreover, incomplete lineage sorting and introgression after hybridization with close relatives could also increase the genetic distance between lineages in different regions (e.g., *F. insipida* and *F. adhatodifolia*; da Costa et al., 2017).

### 4.2 Common genetic patterns

A regional signal of low overall genetic diversity in SWA for the plastid marker was observed in *J. digitata*, and to a lesser degree for *O. parvifolia/glycycarpa* and *F. insipida*. This regional pattern is consistent with the postglacial rain forest expansion reported on the southern margin of the Amazon rain forest in Bolivia, based on pollen and charcoal records (Burbridge, Mayle, & Killeen, 2004; Mayle et al., 2000). Those studies suggested that changes in the position of the Intertropical Convergence Zone (ITCZ) drove subtle shifts in the seasonally dry climate in southwestern Amazonia, with millennial latitudinal migration of the southernmost position of the ITCZ explained by Milankovitch cycles. Thus, historical changes in climate in this part of Amazonia may have favoured the expansion of rain forest during interglacial periods and the restriction of the wet tropical flora to northwestern regions during periods of greater seasonality of rainfall coincident with glacial maxima.

Our results showed lower overall genetic diversity in *J. digitata* and *F. insipida* compared to the shade‐tolerant species. Lowe et al. (2018) suggested that neotropical pioneer species accumulate lower genetic diversity within populations and greater genetic diversity among populations than late successional species due to the loss of genetic diversity by bottlenecks. However, our results contrast with those of Lowe et al. (2018) in that we found less spatial genetic structure in pioneers than shade‐tolerant species. This may be due to the use of different markers: AFLP in the study of Lowe et al. (2018) and *trn*H‐*psb*A here. Moreover, we highlight the presence of a dominant and widespread plastid haplotype in SWA for our two pioneer species. This pattern was also found in other pioneer species such as *Ceiba pentandra* across Amazonia (Dick, Bermingham, Lemes, & Gribel, 2007) and *Cordia alliodora* across the Neotropics (Rymer, Dick, Vendramin, Buonamici, & Boshier, 2013).

The two pioneer species also showed low genetic diversity and no genetic differentiation among populations for ITS compared to shade‐tolerant species. The lack of genetic diversity for ITS may reflect the slow rate of evolution of this region (Dick, Abdul‐Salim, & Bermingham, 2003), but this pattern was only observed in some of the species studied and this could be suggestive of effective nuclear gene flow via pollen. Pollen of *J. digitata* and *F. insipida* is dispersed by sphingid moths (Bawa, Bullock, Perry, Coville, & Grayum, 1985) and tiny aganoid wasps (Machado, Jousselin, Kjellberg, Compton, & Herre, 2001), respectively. Sphingid moths are large in size and have been shown to fly over 300 m between flowering trees in the Neotropics (*Pithecellobium elegans*; Chase, Moller, Kesseli, & Bawa, 1996). Wasps that pollinate monoecious fig species such as *F. insipida* have been reported to travel over distances of up to 14 km in the Neotropics (Nason, Herre, & Hamrick, 1998) and over more than 150 km in riparian vegetation of the Namibian desert (Ahmed, Compton, Butlin, & Gilmartin, 2009). In these two species, pollen dispersal may succeed even among geographically distant individuals, indicating high effective population sizes and gene flow over large distances (Loveless & Hamrick, 1984). Very low genetic diversity in ITS was also characteristic of other pioneer species such as *C. pentandra* (Dick et al., 2007), *C. alliodora* (Rymer et al., 2013), and *Schizolobium parahyba* (Turchetto‐Zolet et al., 2012), in which pollen is dispersed by bats, moths, or bees, respectively (Bawa, Bullock, et al., 1985; Bawa, Perry, & Beach, 1985), and in the shade‐tolerant species *Symphonia globulifera* (Dick & Heuertz, 2008), in which pollen is dispersed by hummingbirds (Bawa, Bullock, et al., 1985). Further studies on the effectiveness of gene flow will require sampling of species with different growth strategies.

## 5 CONCLUSIONS

This comparative phylogeographic study of trees in western Amazonia demonstrates that genetic breaks are not congruent among species. We found no evidence consistent with the effects of Pleistocene refugia or paleoarches. These results imply that Amazon forest trees did not evolve, disperse, and successfully occupy the Amazon environment *en masse*, but rather each individual lineage did so idiosyncratically. The one signature of genetic concordance that we uncovered is that of recent, rapid population expansion in southwestern Amazonia, which is consistent with paleoecological evidence for the expansion of the southern fringe of the Amazonian rain forest since the Last Glacial Maximum (Burbridge et al., 2004; Mayle et al., 2000).

The lack of congruent patterns of genetic structure is consistent with studies that suggest a key role for idiosyncratic historical dispersal in shaping the biogeography of Amazonian tree species (e.g., Dexter et al., 2017). In addition, genetic patterns shown by pioneer and shade‐tolerant species suggest that understanding the contemporary ecology of species is critical to explaining their phylogeography, although our limited sample size in terms of numbers of species precludes formal tests of this (cf. Lowe et al., 2018).

In sum, our results suggest that idiosyncratic dispersal and ecological differences among species together played key roles in shaping patterns of genetic diversity in neotropical rain forest tree species. This would explain why this comparative phylogeographic study, and most prior studies of individual species (e.g., Dick et al., 2007; Dick & Heuertz, 2008; Rymer et al., 2013), did not detect congruent patterns. Like Amazonian birds (Smith et al., 2014), Amazonian tree species appear to have originated and occupied environments individualistically rather than as congruent components of a coherent Amazonian flora.

## CONFLICT OF INTEREST

The authors have no conflict of interest to declare.

## AUTHOR CONTRIBUTIONS

E.H. and R.T.P. conceived the idea; E.H. and K.D. collected the samples; E.H. and M.H. carried out the DNA laboratory work; E.H. performed statistical analyses; E.H., R.T.P., and K.D. wrote the paper; and M.H and O.P. contributed to the writing. All authors read and approved the final manuscript.

## Data Availability

These sequence data have been submitted to the GenBank database under accession numbers KJ734295 ‐ KJ734476 & MK913904 ‐ MK914395 (*trn*H‐*psb*A) and KJ734485 ‐ KJ734561 & MK914396 ‐ MK914532 (ITS).

## References

[ece35306-bib-0001] Ahmed, S. , Compton, S. G. , Butlin, R. K. , & Gilmartin, P. M. (2009). Wind‐borne insects mediate directional pollen transfer between desert fig trees 160 kilometers apart. Proceedings of the National Academy of Sciences, 106, 20342–20347. 10.1073/pnas.0902213106 PMC2787140 19910534

[ece35306-bib-0002] Anhuf, D. , Ledru, M. P. , Behling, H. , Da Cruz Jr, F. W. , Cordeiro, R. C. , Van der Hammen, T. , Karmann, I. , Marengo, J. A. , De Oliveira, P. E. , Pessenda, L. , Siffedine, A. , Albuquerque, A. L. , … Da Silva Dias, P. L. (2006). Paleo‐environmental change in Amazonian and African rainforest during the LGM. Palaeogeography, Palaeoclimatology, Palaeoecology, 239, 510–527. 10.1016/j.palaeo.2006.01.017

[ece35306-bib-0003] Arruda, D. M. , Fernandes‐Filho, E. I. , Solar, R. R. , & Schaefer, C. E. (2017). Combining climatic and soil properties better predicts covers of Brazilian biomes. The Science of Nature, 104, 32 10.1007/s00114-017-1456-6 28324174

[ece35306-bib-0004] Avise, J. C. , Arnold, J. , Ball, R. M. , Bermingham, E. , Lamb, T. , Neigel, J. E. , … Saunders, N. C. (1987). Intraspecific phylogeography: The mitochondrial‐DNA bridge between population‐genetics and systematics. Annual Review of Ecology and Systematics, 18, 489–522. 10.1146/annurev.es.18.110187.002421

[ece35306-bib-0005] Bawa, K. S. , Bullock, S. H. , Perry, D. R. , Coville, R. E. , & Grayum, M. H. (1985). Reproductive biology of tropical lowland rain forest trees. II. Pollination Systems. American Journal of Botany, 72, 346–356. 10.1002/j.1537-2197.1985.tb05358.x

[ece35306-bib-0006] Bawa, K. S. , Perry, D. R. , & Beach, J. H. (1985). Reproductive biology of tropical lowland rain forest trees. I. Sexual systems and incompatibility mechanisms. American Journal of Botany, 72, 331–345. 10.1002/j.1537-2197.1985.tb05357.x

[ece35306-bib-0007] Burbridge, R. E. , Mayle, F. E. , & Killeen, T. J. (2004). Fifty‐thousand‐year vegetation and climate history of Noel Kempff Mercado National Park, Bolivian Amazon. Quaternary Research, 61, 215–230. 10.1016/j.yqres.2003.12.004

[ece35306-bib-0008] Chase, M. R. , Moller, C. , Kesseli, R. , & Bawa, K. S. (1996). Distant gene flow in tropical trees. Nature, 383, 398–399. 10.1038/383398a0

[ece35306-bib-0009] Cheng, H. , Sinha, A. , Cruz, F. W. , Wang, X. F. , Edwards, R. L. , dHorna, F. M. , … Auler, A. S. (2013). Climate Change Patterns in Amazonia and Biodiversity. Nature Communications, 4, 1411 10.1038/ncomms2415 23361002

[ece35306-bib-0010] Colinvaux, P. A. , De Oliveira, P. E. , & Bush, M. B. (2000). Amazonian and Neotropical plant communities on glacial time‐scales: The failure of the aridity and refuge hypotheses. Quaternary Science Reviews, 19, 141–169. 10.1016/S0277-3791(99)00059-1

[ece35306-bib-0011] da Costa, P. C. , Lorenz‐Lemke, A. P. , Furini, P. R. , Honorio Coronado, E. N. , Kjellberg, F. , & Pereira, R. A. (2017). The phylogeography of two disjunct Neotropical Ficus (Moraceae) species reveals contrasted histories between the Amazon and the Atlantic Forests. Botanical Journal of the Linnean Society, 185, 272–289. 10.1093/botlinnean/box056

[ece35306-bib-0012] da Silva, M. N. F. , & Patton, J. L. (1998). Molecular phylogeography and the evolution and conservation of Amazonian mammals. Molecular Ecology, 7, 475–486. 10.1046/j.1365-294x.1998.00276.x 9628001

[ece35306-bib-0013] Dauby, G. , Duminil, J. , Heuertz, M. , Koffi, G. , Stevart, T. , & Hardy, O. J. (2014). Congruent phylogeographical patterns of eight tree species in Atlantic Central Africa provide insights into the past dynamics of forest cover. Molecular Ecology, 23, 2299–2312. 10.1111/mec.12724 24655106

[ece35306-bib-0014] de Fátima Rossetti, D. , Mann de Toledo, P. , & Góes, A. M. (2005). New geological framework for Western Amazonia (Brazil) and implications for biogeography and evolution. Quaternary Research, 63, 78–89. 10.1016/j.yqres.2004.10.001

[ece35306-bib-0015] Dexter, K. G. , Lavin, M. , Torke, B. M. , Twyford, A. D. , Kursar, T. A. , Coley, P. D. , Drake, C. , Hollands, R. , … Pennington, R. T. (2017). Dispersal assembly of rain forest tree communities across the Amazon basin. Proceedings of the National Academy of Sciences USA, 114, 2645–2650. 10.1073/pnas.1613655114 PMC5347625 28213498

[ece35306-bib-0016] Dexter, K. G. , Terborgh, J. W. , & Cunningham, C. W. (2012). Historical effects on beta diversity and community assembly in Amazonian trees. Proceedings of the National Academy of Sciences USA, 109, 7787–7792. 10.1073/pnas.1203523109 PMC3356654 22547831

[ece35306-bib-0017] Dick, C. W. , Abdul‐Salim, K. , & Bermingham, E. (2003). Molecular systematic analysis reveals cryptic tertiary diversification of a widespread tropical rain forest tree. American Naturalist, 162, 691–703. 10.1086/379795 14737707

[ece35306-bib-0018] Dick, C. W. , Bermingham, E. , Lemes, M. R. , & Gribel, R. (2007). Extreme long‐distance dispersal of the lowland tropical rainforest tree *Ceiba pentandra* L. (Malvaceae) in Africa and the Neotropics. Molecular Ecology, 16, 3039–3049. 10.1111/j.1365-294X.2007.03341.x 17614916

[ece35306-bib-0019] Dick, C. W. , & Heuertz, M. (2008). The complex biogeographic history of a widespread tropical tree species. Evolution, 62, 2760–2774. 10.1111/j.1558-5646.2008.00506.x 18764917

[ece35306-bib-0020] Dick, C. W. , Lewis, S. L. , Maslin, M. , & Bermingham, E. (2013). Neogene origins and implied warmth tolerance of Amazon tree species. Ecology and Evolution, 3, 162–169. 10.1002/ece3.441 PMC3568851 23404439

[ece35306-bib-0021] Doyle, J. J. , & Doyle, J. L. (1987). A rapid DNA isolation procedure for small quantities of fresh leaf tissue. Phytochemical Bulletin, 19, 11–15.

[ece35306-bib-0022] Dupanloup, I. , Schneider, S. , & Excoffier, L. (2002). A simulated annealing approach to define the genetic structure of populations. Molecular Ecology, 11, 2571–2581. 10.1046/j.1365-294X.2002.01650.x 12453240

[ece35306-bib-0023] Espurt, N. , Baby, P. , Brusset, S. , Roddaz, M. , Hermoza, W. , & Barbarand, J. (2010). The Nazca Ridge and uplift of the Fitzcarrald Arch: Implications for regional geology in northern South America In Hoorn C. & Wesselingh F. P. (Eds.), Amazonia, landscape and species evolution: A look into the past (pp. 89–100). Oxford, UK: Blackwell‐Wiley.

[ece35306-bib-0024] Excoffier, L. , & Lischer, H. E. L. (2010). Arlequin suite ver 3.5: A new series of programs to perform population genetics analyses under Linux and Windows. Molecular Ecology Resources, 10, 564–567. 10.1111/j.1755-0998.2010.02847.x 21565059

[ece35306-bib-0025] Fu, Y. X. (1997). Statistical tests of neutrality of mutations against population growth, hitchhiking and background selection. Genetics, 147, 915–925. 9335623 10.1093/genetics/147.2.915 PMC1208208

[ece35306-bib-0026] Harpending, H. (1994). Signature of ancient population growth in a low‐resolution mitochondrial DNA mismatch distribution. Human Biology, 66, 591–600. 8088750

[ece35306-bib-0027] Heuertz, M. , Duminil, J. , Dauby, G. , Savolainen, V. , & Hardy, O. J. (2014). Comparative phylogeography in rainforest trees from Lower Guinea, Africa. PLoS ONE, 9, e84307 10.1371/journal.pone.0084307 24416215 PMC3885573

[ece35306-bib-0028] Higgins, M. A. , Ruokolainen, K. , Tuomisto, H. , Llerena, N. , Cardenas, G. , Phillips, O. L. , … Räsänen, M. (2011). Geological control of floristic composition in Amazonian forests. Journal of Biogeography, 38, 2136–2149. 10.1111/j.1365-2699.2011.02585.x 22247585 PMC3253337

[ece35306-bib-0029] Honorio Coronado, E. N. , Dexter, K. G. , Poelchau, M. F. , Hollingsworth, P. M. , Phillips, O. L. , & Pennington, R. T. (2014). *Ficus insipida* subsp. insipida (Moraceae) reveals the role of ecology in the phylogeography of widespread Neotropical rain forest tree species. Journal of Biogeography, 41, 1697–1709. 25821341 10.1111/jbi.12326 PMC4368618

[ece35306-bib-0030] Jones, F. A. , Cerón‐Souza, I. , Hardesty, B. D. , & Dick, C. W. (2013). Genetic evidence of Quaternary demographic changes in four rain forest tree species sampled across the Isthmus of Panama. Journal of Biogeography, 40, 720–731. 10.1111/jbi.12037

[ece35306-bib-0031] Lougheed, S. C. , Gascon, C. , Jones, D. A. , Bogart, J. P. , & Boag, P. T. (1999). Ridges and rivers: A test of competing hypotheses of Amazonian diversification using a dart‐poison frog (*Epipedobates femoralis*). Proceedings of the Royal Society of London. Series B: Biological Sciences, 266, 1829–1835. 10535104 10.1098/rspb.1999.0853 PMC1690219

[ece35306-bib-0032] Loveless, M. D. , & Hamrick, J. L. (1984). Ecological determinants of genetic structure in plant populations. Annual Review of Ecology and Systematics, 15, 65–95. 10.1146/annurev.es.15.110184.000433

[ece35306-bib-0033] Lowe, A. J. , Breed, M. F. , Caron, H. , Colpaert, N. , Dick, C. , Finegan, B. , … Cavers, S. (2018). Standardized genetic diversity‐life history correlates for improved genetic resource management of Neotropical trees. Diversity and Distributions, 24, 730–741. 10.1111/ddi.12716

[ece35306-bib-0034] Machado, C. A. , Jousselin, E. , Kjellberg, F. , Compton, S. G. , & Herre, E. A. (2001). Phylogenetic relationships, historical biogeography and character evolution of fig‐pollinating wasps. Proceedings of the Royal Society of London. Series B: Biological Sciences, 268, 685–694. 11321056 10.1098/rspb.2000.1418 PMC1088657

[ece35306-bib-0035] Maddison, W. P. , & Maddison, D. R. (2001) Mesquite: A modular system for evolutionary analysis version 2.74.

[ece35306-bib-0036] Mayle, F. E. , Burbridge, R. , & Killeen, T. J. (2000). Millennial‐scale dynamics of southern Amazonian rain forests. Science, 290, 2291–2294. 10.1126/science.290.5500.2291 11125139

[ece35306-bib-0037] Motamayor, J. C. , Lachenaud, P. , da Silva e Mota, J. W. , Loor, R. , Kuhn, D. N. , Brown, J. S. , & Schnell, R. J. (2008). Geographic and genetic population differentiation of the Amazonian chocolate tree (*Theobroma cacao* L). PLoS ONE, 3, e3311 10.1371/journal.pone.0003311 18827930 PMC2551746

[ece35306-bib-0038] Nason, J. D. , Herre, E. A. , & Hamrick, J. L. (1998). The breeding structure of a tropical keystone plant resource. Nature, 391, 685–687. 10.1038/35607

[ece35306-bib-0039] Paradis, E. (2010). Pegas: An R package for population genetics with an integrated–modular approach. Bioinformatics, 26, 419–420. 10.1093/bioinformatics/btp696 20080509

[ece35306-bib-0040] Petit, R. J. , Aguinagalde, I. , de Beaulieu, J. L. , Bittkau, C. , Brewer, S. , Cheddadi, R. , … Vendramin, G. G. (2003). Glacial refugia: Hotspots but not melting pots of genetic diversity. Science, 300, 1563–1565. 10.1126/science.1083264 12791991

[ece35306-bib-0041] Poelchau, M. F. , & Hamrick, J. L. (2013). Comparative phylogeography of three common Neotropical tree species. Journal of Biogeography, 40, 618–631. 10.1111/j.1365-2699.2011.02599.x

[ece35306-bib-0042] Pons, O. , & Petit, R. J. (1996). Measuring and testing genetic differentiation with ordered versus unordered alleles. Genetics, 144, 1237–1245. 8913764 10.1093/genetics/144.3.1237 PMC1207615

[ece35306-bib-0043] Prance, G. T. (1982). Biological diversification in the tropics. New York, NY: Columbia University.

[ece35306-bib-0044] Räsänen, M. E. , Salo, J. S. , Jungnert, H. , & Pittman, L. R. (1990). Evolution of the western Amazon lowland relief: Impact of Andean foreland dynamics. Terra Nova, 2, 320–332. 10.1111/j.1365-3121.1990.tb00084.x

[ece35306-bib-0045] Regard, V. , Lagnous, R. , Espurt, N. , Darrozes, J. , Baby, P. , Roddaz, M. , … Hermoza, W. (2009). Geomorphic evidence for recent uplift of the Fitzcarrald Arch (Peru): A response to the Nazca Ridge subduction. Geomorphology, 107, 107–117. 10.1016/j.geomorph.2008.12.003

[ece35306-bib-0046] Rogers, A. R. , & Harpending, H. (1992). Population growth makes waves in the distribution of pairwise genetic differences. Molecular Biology and Evolution, 9, 552–569. 1316531 10.1093/oxfordjournals.molbev.a040727

[ece35306-bib-0048] Rymer, P. D. , Dick, C. W. , Vendramin, G. G. , Buonamici, A. , & Boshier, D. (2013). Recent phylogeographic structure in a widespread ‘weedy’ Neotropical tree species, *Cordia alliodora* (Boraginaceae). Journal of Biogeography, 40, 693–706.

[ece35306-bib-0049] Schneider, S. , & Excoffier, L. (1999). Estimation of past demographic parameters from the distribution of pairwise differences when the mutation rates vary among sites: Application to human mitochondrial DNA. Genetics, 152, 1079–1089. 10388826 10.1093/genetics/152.3.1079 PMC1460660

[ece35306-bib-0050] Shaw, J. , Lickey, E. B. , Schilling, E. E. , & Small, R. L. (2007). Comparison of whole chloroplast genome sequences to choose noncoding regions for phylogenetic studies in angiosperms: The tortoise and the hare III. American Journal of Botany, 94, 275–288. 10.3732/ajb.94.3.275 21636401

[ece35306-bib-0051] Smith, B. T. , McCormack, J. E. , Cuervo, A. M. , Hickerson, M. J. , Aleixo, A. , Cadena, C. D. , … Brumfield, R. T. (2014). The drivers of tropical speciation. Nature, 515, 406–409. 10.1038/nature13687 25209666

[ece35306-bib-0052] Turchetto‐Zolet, A. C. , Cruz, F. , Vendramin, G. G. , Simon, M. F. , Salgueiro, F. , Margis‐Pinheiro, M. , & Margis, R. (2012). Large‐scale phylogeography of the disjunct Neotropical tree species *Schizolobium parahyba* (Fabaceae‐Caesalpinioideae). Molecular Phylogenetics and Evolution, 65, 174–182. 10.1016/j.ympev.2012.06.012 22750114

[ece35306-bib-0053] White, T. J. , Bruns, T. , Lee, S. , & Taylor, J. (1990). Amplification and direct sequencing of fungal ribosomal RNA genes for phylogenetics In Innis M. A., Gelfand D. H., Shinsky J. J.…White T. J. (Eds.), PCR protocols: A guide to methods and applications (pp. 315–322). San Diego, CA: Academic Press.

